# Associations among Household Animal Ownership, Infrastructure, and Hygiene Characteristics with Source Attribution of Household Fecal Contamination in Peri-Urban Communities of Iquitos, Peru

**DOI:** 10.4269/ajtmh.20-0810

**Published:** 2020-11-02

**Authors:** Francesca Schiaffino, Dixner Rengifo Trigoso, Josh M. Colston, Maribel Paredes Olortegui, Wagner V. Shapiama Lopez, Paul F. Garcia Bardales, Nora Pisanic, Meghan F. Davis, Pablo Penataro Yori, Margaret N. Kosek

**Affiliations:** 1 Department of International Health, Johns Hopkins Bloomberg School of Public Health, Baltimore, Maryland;; 2 Faculty of Veterinary Medicine, Universidad Peruana Cayetano Heredia, Lima, Peru;; 3 Asociacion Benefica Prisma, Iquitos, Peru;; 4 Division of Infectious Diseases and International Health, University of Virginia School of Medicine, Charlottesville, Virginia;; 5 Department of Environmental Health and Engineering, Johns Hopkins Bloomberg School of Public Health, Baltimore, Maryland;; 6 Department of Molecular and Comparative Pathobiology, Johns Hopkins Bloomberg School of Medicine, Baltimore Maryland

## Abstract

Using previously validated microbial source tracking markers, we detected and quantified fecal contamination from avian species and avian exposure, dogs, and humans on household cooking tables and floors. The association among contamination, infrastructure, and socioeconomic covariates was assessed using simple and multiple ordinal logistic regressions. The presence of *Campylobacter* spp. in surface samples was linked to avian markers. Using molecular methods, animal feces were detected in 75.0% and human feces in 20.2% of 104 households. Floors were more contaminated than tables as detected by the avian marker *Av4143*, dog marker *Bactcan*, and human marker *Bachum*. Wood tables were consistently more contaminated than non-wood surfaces, specifically with the mitochondrial avian markers *ND5* and CytB, fecal marker Av4143, and canine marker *Bactcan*. Final multivariable models with socioeconomic and infrastructure characteristics included as covariates indicate that detection of avian feces and avian exposure was associated with the presence of chickens, maternal age, and length of tenancy, whereas detection of human markers was associated with unimproved water source. Detection of *Campylobacter* in surface samples was associated with the avian fecal marker Av4143. We highlight the critical need to detect and measure the burden of animal fecal waste when evaluating household water, hygiene, and sanitation interventions, and the possibility of decreasing risk of exposure through the modification of surfaces to permit more effective household disinfection practices. Animals may be a more important source of household fecal contamination than humans in many low-resource settings, although interventions have historically focused almost exclusively on managing human waste.

## INTRODUCTION

Domestic animal husbandry at the household level is a common practice in low-resource rural communities, contributing to intra-domiciliary fecal contamination and potentially increasing the risk of transmission of zoonotic enteric pathogens.^1^ Water, soil, and household surfaces are regularly contaminated with bacterial pathogens of both human and other animal fecal material.^2–5^ Detection and quantification of fecal contamination within the house is generally carried out using standard microbiologic methods targeting traditional fecal indicator bacteria (FIB), such as *Escherichia coli*. Fecal indicator bacteria are commonly detected and quantified in household water and surfaces, and epidemiologic studies have associated the degree of fecal contamination with hygiene, sanitation infrastructure, and sociodemographic characteristics of household members without accounting for the production of FIB from domestic and peri-domestic animal sources.^4,6,7^ One of the principal assumptions underpinning these studies is that fecal contamination is mainly human derived. However, animal fecal matter is often also highly prevalent within households, and the presence of animals, as well as animal fecal waste, has been associated with increased risk of enteric illness.^8–10^ Although traditional FIB are able to determine the degree of fecal contamination by taking into consideration all sources, these methods are not capable of distinguishing between animal and human sources of detected feces, and this fundamentally stifles directed control measures.^11,12^ Furthermore, samples analyzed using traditional microbiologic methods are easily contaminated by FIB from other environmental sources or reservoirs, such as soils.^13,14^

Microbial source tracking (MST) methods have been developed to determine the source of fecal contamination within wastewater and recreational water systems for remediation purposes.^15,16^ By attributing and quantifying fecal contamination to a specific animal species, intervention strategies and remediation measures can be more easily targeted and applied. Several MST markers have been developed and standardized in rural communities where there is a high degree of household fecal contamination.^17–21^ However, to date and to our knowledge, no studies have applied MST methods to quantify animal fecal burden in household surfaces. Furthermore, few studies that have applied MST markers in low-income settings have included avian-specific markers of fecal contamination.^22–24^ This is of critical importance, given the role of poultry within domestic animal husbandry practices as an alternative source of income and nutritional source of protein, as well as a source of *Campylobacter* spp., one of the main causes of bacterial diarrhea, stunting, and environmental enteropathy in pediatric populations of low-income settings.^25–28^

Using MST markers previously validated in the same region, this study quantified the burden of animal fecal contamination of surfaces by animal species, in households in a peri-urban, low-resource, tropical community of Loreto, Peru, with a particular emphasis on avian fecal contamination.^29^ We assessed the associations between MST presence and burden in household surfaces with household infrastructure and socioeconomic characteristics of the primary caregiver. Finally, we assess the association between the detection of *Campylobacter* spp. and different MST markers in surface samples from the same households.

## MATERIALS AND METHODS

### Study setting and population.

This study was carried out in Santa Clara de Nanay, Santo Tomas, and La Union (3°47′S, 73°20′W), three peri-urban communities located 15 km away from Iquitos city center, Loreto, Peru. These communities combined are composed of approximately 1,300 households and 10,000 individuals. Common occupations for men in these communities include small-scale agricultural production, fishing, and moto-taxi driving, whereas women most commonly report being homemakers, having a small corner shop (“bodega”) or being unemployed.^30^

### Data and sample collection.

Between October 2018 and September 2019, households from these three communities were randomly selected for sampling. Within each household, two surface samples were obtained, and a socioeconomic questionnaire was completed by the head of the household. Surfaces sampled included the main table where cooking took place and entrance floors, for reasons described previously.^4^ The cooking surface of preference (where food is manipulated for human consumption) was identified by the head of the household. As described previously, a 30.0-cm by 30.0-cm square of scrap paper used to frame the sampling area was placed on top of the selected surface.^6,31^ Using sterile nitrile gloves and applying moderate pressure, half of a dry autoclaved electrostatic cloth (Swiffer^®^, Procter & Gamble, Cincinnati, OH) was spread over the framed surface area. The cloth was then placed in a sterile 24-oz Whirl-Pak bag (Nasco, WI) and 10 mL of phosphate-buffered solution (PBS) added. Samples were placed in a cooler with ice-packs and transported to the laboratory within 4–6 hours of collection for processing.

If there was a child younger than 2 years present in the household, a plastic toy was given and exchanged for an identical item within 24 hours as a sentinel object that reports more directly on the microbial exposure of the mobile child.^32^ The toy was placed in a plastic bag, and 10 mL of PBS were added. All samples went through the same processing protocol. This sampling strategy was inspired by the work of Vujcic et al.^32^ in Bangladesh.

### Sample processing.

Samples were vigorously shaken for 5 minutes to ensure the sampled material was homogenized within the PBS solution. The solution was transferred into sterile 2 mL crioviales for DNA extraction. DNA was extracted from 500 mL of solution using PowerSoil^®^ DNA extraction kit (Qiagen, Germantown, MD) following bead-beating according to the manufacturer’s instructions. For each extraction, a negative control consisting of RNA-free water was used.

### Quantitative polymerase chain reaction using microbial source tracking markers.

Eight MST markers that have previously been validated within this context were used to score surface samples.^29^ Specifically, two avian fecal markers (*Av4143* and *LA35*), two avian mitochondrial fecal markers (*cytb* and *ND5*), two human fecal markers (*BachHum* and *HF83-Taqman*), one dog fecal marker (*BactCan*), and one pig fecal marker (*Pig2Bac*) were used.^33–37^ Details regarding the target species, gene, and internal validity parameters of all eight MST markers are presented in Supplemental Table 1.

TaqMan assays consisted of final reaction mixtures of 20 µL, which included TaqMan^™^ Advanced Fast Start Master Mix (×2) (Applied Biosystems, Foster City, CA), forward and reverse primers (200 µM), probes (100 µM), 5 µL of DNA template, and RNA-free water (Ambion^™^, Thermo Fisher Scientific, Waltham, MA). Negative controls consisting of RNA- and DNA-free water were included in each amplification reaction. Reaction mixtures were placed in a 96-well plate and amplified using a StepOnePlus real-time PCR system (Applied Biosystems). Internal amplification controls (qHsaCtlP0001003, Bio-Rad Laboratories, Irvine, CA) were run for every marker and surface sample, and runs were invalid if the internal standard did not amplify. Standard amplification conditions (95°C for 5 minutes, 40 cycles of 95°C for 15 seconds, 53°C for 15 seconds, and 60°C for 45 seconds) were used for all reactions, except for *LA35* and *Av4143*, for which annealing temperatures were set at 56°C and 55°C, respectively. Standard curve analysis was performed as reported previously.^29^

### Detection of *Campylobacter* spp.

Surface samples were tested for the presence or absence of *Campylobacter* spp. using a semi-quantitative PCR that targeted a 16S sRNA segment that identifies all members of the *Campylobacter* genus^38^ as well as the cadF gene (adhesion to fibronectin) to detect thermotolerant species (most likely *Campylobacter jejuni*/*Campylobacter coli* only^39^) (Supplemental Table 2). Final reaction mixtures of 25 µL consisted of 12.5 µL of Taq Environmental Master Mix 2.0 (Thermo Fisher Scientific), primers at a concentration of 0.2 µM each, and the probes at a concentration of 0.1 µM each, 1 µL of DNA, and nuclease-free water. The assay was performed under the following cycling conditions: 95°C for 10 minutes, 45 cycles of 95°C for 15 seconds, and 55°C for 1 minute (StepOne Instrument, Applied Biosystems). A target was determined to be positive if a cycle threshold (Ct) of less than 38 was obtained for the 16S gene.

### Data analysis.

A binary variable indicating the presence and absence of a given MST marker in a surface sample was created using the assay-specific limit of detection, indicating a positive sample if the Ct obtained was below the limit of detection (lower Ct) and a negative sample if the Ct was above that limit or was undetermined. Determination of assay-specific limits of detection has been described previously.^29^ Floor and table surface samples were treated as distinct independent samples. Floor was categorized as either finished—made of a material that separated the dirt floor from household members or animals—or unfinished—uncovered earth.^40^ The surface material of tables was classified as being either of wood or non-wood, the latter category mainly composed of wood tables covered with plastic sheeting. Pearson’s chi-square and Fisher’s exact test were used to test the differences in positivity for any specific MST marker between floors and tables, as well as between unfinished and finished floors, and wood and non-wood tables. Gene quantities were log_10_ transformed (log_10_ gene copy number [GCN]/µL). The distribution of this continuous variable was assessed using Shapiro–Wilk, skewness, and kurtosis normality tests. Data were found to be right skewed for all six markers. The difference in the median log_10_ GCN/µL in tables and floors, as well as unfinished versus finished floors and non-wood versus wood floors was assessed using the Wilcoxon rank-sum test. Gene quantities in floor and table samples were independently categorized into tertiles and modeled as an ordinal outcome variable, where the first tertile was interpreted as “low,” the second as “medium,” and the third one as “high” GCN quantities.

Covariates analyzed included age of the primary caregiver (years), maternal education (years), age of the primary caregiver at first pregnancy (years), and average monthly income (US dollars). Household infrastructure characteristics included a binary variable for household crowding (less than six people living in the household/more than six people living in the household), length of household tenancy (less than 1 year, between 1 and 5 years, between 5 and 10 years, between 10 and 20 years, and more than 20 years), floor material (unfinished/finished), table material (wood/non-wood), and wall type (cement/other). Hygiene covariates included treatment of drinking water, water source (improved/unimproved), and sanitation facility (improved/unimproved), as defined by the WHO/UNICEF Joint Monitoring Program for Water Supply and Sanitation.^41^ Finally, the presence and absence of chickens within the household at the time of visit were also included as a covariate.

Baseline associations among the main exposures, floor material, and table material were performed using Pearson’s chi-square or Fisher’s exact test for binary covariates and Wilcoxon rank-sum test for continuous covariates. Regression models were fitted for all MST markers separately using simple and multiple ordered logistic regressions to test the association between the degree of contamination (“low,” “medium,” or “high”) in floor and table samples, and the specified household and socioeconomic covariates. Multivariable regression models were fitted with all household and socioeconomic covariates. The proportional odds assumption was tested for all adjusted and unadjusted regression models.

Associations between MST marker detection and the presence of *Campylobacter* in floor and table samples were assessed by logistic regression with the presence of *Campylobacter* as the binary outcome variable and using the odds ratio (OR) as the measure of effect. Separate models were run for each MST marker, and the presence of chickens in the household was included as a covariate in all models. Type I error was set at 0.05 for all statistical analyses. Data management and statistical analysis were performed in STATA 14 and (Stata Corp., CollegeStation, TX) and R (version 3.3.2, R Foundation for Statistical Computing, Vienna, Austria).

### Ethical considerations.

Ethical approval was obtained from the international review boards of Asociacion Benefica Prisma and Johns Hopkins Bloomberg School of Public Health. A local field-worker from Asociacion Benefica Prisma explained the study procedures to the household head, and signed informed consent was requested before any study procedure was initiated.

## RESULTS

**Figure 1.**
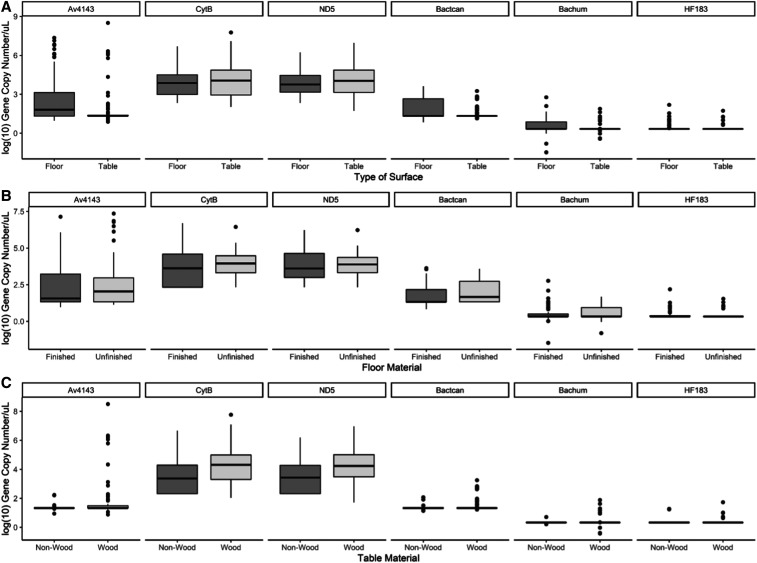
Quantitative burden of microbial source tracking markers in (**A**) floor and table samples, (**B**) unfinished and finished floors, and (**C**) wood and non-wood samples from households in three communities of Iquitos, Peru. * unfinished = dirt floor surface samples; finished = cement, tile, wood, or plastic floor surface samples; non-wood = tile, plastic, paper, or cloth table surface samples. Higher burden of animal feces in comparison to human feces in table and wood surfaces. Statistically significant higher burden of avian mitochondrial DNA and dog feces in floors in comparison to tables.

A total of 104 samples obtained from surfaces used for food preparation and 104 matched floor samples were obtained from 104 households. Of the 104 floor samples, 54 (51.9%) were made of dirt, 39 (37.5%) of cement, six (5.8%) of wood, four (3.8%) of tile, and one (1.0%) floor was covered in plastic material. A total of 54 floors were classified as unfinished (i.e., bare earth), and 50 were classified as finished. Of the 104 table samples, 75 (72.1%) were made of wood, 22 (21.2%) of plastic, four (3.8%) of fabric, two (1.9%) of tile, and one (1.0%) was covered in paper (Table 1). At least 76.9% (80/104) of households were positive for any fecal marker (*Av4143, Bactcan*, *BacHum*, and *HF183-Taqman*) in either floor or table samples. Animal feces were detected in 75% (78/104) of households, and human feces were detected in 20.2% (21/104) of households. Bivariate results of each MST marker by table and floor surface categories are shown in Table 2, quantitative results are shown in Figure 1, and categorical (high, medium, and low data) results for each MST marker are shown in Supplemental Table 3. All samples were negative for the avian fecal marker *LA35* and pig fecal marker *Pig2Bac*. Covariate characteristics of household and primary caregiver are presented in Table 3. The univariate associations between MST gene quantity tertiles of floors and tables surface samples, and the household and primary caregiver covariates are shown in the Supplemental Material (Supplemental Tables 4 and 5), whereas the adjusted associations are presented in Table 4.

**Table 1:** Materials of floor and table surface samples from households (*N* = 104) in three communities of Iquitos, Loreto, Peru

Material	Surfaces (*n* = 208)	
	Floors (*n* = 104), *n* (%)	Tables (*n* = 104), *n* (%)
Cement	39 (37.5)	0 (0.0)
Tile	4 (3.8)	2 (1.9)
Plastic	1 (9.6)	22 (21.2)
Dirt	54 (51.9)	0 (0.0)
Wood	6 (5.8)	75 (72.1)
Paper	0 (0.0)	1 (9.6)
Cloth	0 (0.0)	4 (3.8)

**Table 2:** Percent positive (A) floor and table samples, (B) unfinished and finished floors, and (C) wood and non-wood samples from households in three communities of Iquitos, Peru, analyzed using MST markers

MST	Target species	Floors	Tables	*P*-value*
		Percent positive (*n*/*N*)	Percent positive (*n*/*N*)	
*Av4143*	Domestic birds and waterfowl	**49.03 (51/104)**	**15.38 (16/104)**	**< 0.001**
*CytB*	Chickens and ducks	59.62 (62/104)	67.31 (70/104)	0.249
*ND5*	Chickens and ducks	83.65 (87/104)	77.88 (81/104)	0.291
*Bactcan*	Dogs	**27.88 (29/104)**	**11.54 (12/104)**	**0.003**
*Bachum*	Humans	**17.31 (18/104)**	**3.85 (4/104)**	**0.003**
*HF-183 Taqman*	Humans	0.96 (1/104)	0.96 (1/104)	NA

MST marker	Target species	Unfinished floors	Finished floors	*P*-value*
		Percent positive (*n*/*N*)	Percent positive (*n*/*N*)	
*Av4143*	Domestic birds and waterfowl	57.4 (31/54)	40.0 (20/50)	0.082
*CytB*	Chickens and ducks	64.8 (35/54)	54.0 (27/50)	0.319
*ND5*	Chickens and ducks	88.9 (48/54)	78.0 (39/50)	0.185
*Bactcan*	Dogs	35.2 (19/54)	20.0 (10/50)	0.125
*Bachum*	Humans	15.0 (8/54)	20.0 (10/50)	0.606
*HF-183 Taqman*	Humans	0.00 (0/54)	2.0 (1/50)	0.481

MST marker	Target species	Wood tables	Non-wood tables	*P*-value*
		Percent positive (*n*/*N*)	Percent positive (*n*/*N*)	
*Av4143*	Domestic birds and waterfowl	18.7 (14/75)	7.0 (2/29)	0.224
*CytB*	Chickens and ducks	72.0 (54/75)	55.2 (16/29)	0.101
*ND5*	Chickens and ducks	84 (63/75)	62.1 (18/29)	**0.016**
*Bactcan*	Dogs	12.0 (9/75)	10.3 (3/29)	1.000
*Bachum*	Humans	5.3 (4/75)	0.0 (0/29)	0.574
*HF-183 Taqman*	Humans	1.3 (1/75)	0.0 (0/29)	1.000

MST = microbial source tracking. A greater number of floor samples are positive for the presence of avian, dog, and human feces, and a greater number of wood tables are positive for avian mitochondrial DNA in comparison to non-wood tables. Bold values indicate *P*-value < 0.05.

**Table 3:** Female head of household, infrastructure, and hygiene characteristics by floor and table materials from households located in three communities of Iquitos, Peru

Covariate	Floors			Tables		
	Finished (*n* = 50)	Unfinished (*n* = 54)	*P*-value	Wood (*n* = 75)	Non-wood (*n* = 29)	*P*-value
Female head of household characteristics						
Maternal age (years), mean (95% CI)	28.2 (26.1–30.4)	30.5 (27.6–33.3)	0.217	29.0 (26.9–31.1)	30.4 (26.8–34.0)	0.515
Maternal education, mean (95% CI)	8.7 (7.9–9.5)	7.75 (7.04–8.46)	0.085	8.0 (7.3–8.6)	8.9 (8.0–9.8)	0.103
Age of first pregnancy (years), mean (95% CI)	18.6 (17.2–20.1)	17.6 (16.8–18.4)	0.212	17.7 (16.8–18.7)	19.0 (17.4–20.5)	0.175
Monthly income (Peruvian sol), mean (95% CI)	374.0 (333.5–414.5)	314.1 (275.9–352.2)	**0.033**	323.5 (296.3–350.6)	398.3 (327.8–468.8)	**0.016**
Household infrastructure characteristics						
Number of people sleeping in household, mean (SD)	6.4 (5.7–7.0)	5.8 (5.1–6.5)	0.209	6.2 (5.6–6.8)	5.6 (4.8–6.3)	0.216
Length of household tenancy (years), % (*n*/*N*)	–	–	0.228			0.739
Less than 1 (*n* = 12)	6.0 (3/50)	16.7 (9/54)	–	10.7 (8/75)	6.9 (2/29)	–
Between 1 and 5 (*n* = 28)	32.0 (16/50)	22.2 (12/54)	–	29.3 (22/75)	20.7 (6/29)	–
Between 5 and 10 (*n* = 22)	16.0 (8/50)	25.9 (14/54)	–	22.7 (17/75)	20.7 (6/29)	–
Between 10 and 20 (*n* = 14)	14.0 (7/50)	13.0 (7/54)	–	12 (9/75)	17.2 (5/29)	–
More than 20 (*n* = 28)	32.0 (16/50)	22.2 (12/54)	–	25.3 (19/75)	34.5 (10/29)	–
Wall type % (*n*/*N*)	–	–	–	–	–	–
Cement	36 (18/50)	11.1 (6/54)	**0.003**	16 (12/75)	48.3 (14/29)	**< 0.001**
Other	64 (32/50)	88.9 (48/54)		84 (63/75)	51.7 (15/29)	
Hygiene characteristics % (*n*/*N*)						
Household treats drinking water	62.0 (31/50)	61.1 (33/54)	0.926	62.7 (47/75)	58.6 (17/29)	0.704
Water Source	–	–				
Improved	90.0 (45/50)	88.9 (48/54)	0.854	86.7 (65/75)	96.6 (28/29)	0.142
Unimproved	10.0 (5/50)	11.1 (6/54)		13.3 (10/75)	3.4 (1/29)	
Sanitation facility	–	–				
Improved	60.0 (30/50)	38.9 (21/54)	**0.031**	42.7 (32/75)	65.5 (19/29)	**0.037**
Unimproved	40.0 (20/50)	61.1 (33/54)		57.3 (43/75)	34.5 (10/29)	
Chickens in household (*n* = 50)	54 (27/50)	42.6 (23/54)	0.245	49.3 (37/75)	51.7 (15/29)	0.827

**Table 4:** Associations among female head of household, infrastructure, and hygiene characteristics, and the change in concentration tertile of each MST marker among floor and table samples from households in three communities of Iquitos, Peru

	*AV4143*				ND5				CYTB				BactCan				BacHum				HF183-Taqman			
	Floors		Tables		Floors		Tables		Floors		Tables		Floors		Tables		Floors		Tables		Floors		Tables	
	OR (SD)	95% CI	OR (SD)	95% CI	OR (SD)	95% CI	OR (SD)	95% CI	OR (SD)	95% CI	OR (SD)	95% CI	OR (SD)	95% CI	OR (SD)	95% CI	OR (SD)	95% CI	OR (SD)	95% CI	OR (SD)	95% CI	OR (SD)	95% CI
Female head of household characteristics																								
Age (years)	**0.93 (0.03)**	**0.87-0.99**	1.02 (0.04)	0.95–1.10	0.95 (0.03)	0.90–1.00	0.97 (0.03)	0.92–1.02	**0.94 (0.03)**	**0.89-0.99**	0.99 (0.03)	0.94–1.04	1.04 (0.03)	0.98–1.10	1.07 (0.04)	0.99–1.16	1.02 (0.03)	0.96–1.09	**0.81 (0.07)**	**0.68-0.97**	1.02 (0.05)	0.93–1.12	1.03 (0.04)	0.96–1.12
Maternal education (years)	0.98 (0.11)	0.79–1.22	1.06 (0.13)	0.84–1.35	0.86 (0.08)	0.72–1.04	0.98 (0,09)	0.82–1.19	0.85 (0.08)	0.71–1.02	1.02 (0.09)	0.85–1.22	1.06 (0.11)	0.86–1.30	1.19 (0.20)	0.86–1.65	1.06 (0.12)	0.85–1.31	0.76 (0.15)	0.52–1.12	0.96 (0.14)	0.72–1.28	0.79 (0.14)	0.56–1.10
Age of first pregnancy (years)	1.11 (0.07)	0.97–1.26	0.94 (0.07)	0.82–1.07	1.07 (0.06)	0.96–1.20	0.99 (0.05)	0.90–1.11	1.06 (0.06)	0.95–1.19	0.94 (0.05)	0.84–1.05	0.96 (0.06)	0.84–1.08	0.88 (0.09)	0.72–1.06	0.99 (0.07)	0.86–1.13	1.36 (0.21)	0.99–1.84	0.99 (0.09)	0.83–1.18	0.93 (0.11)	0.56–1.10
Average monthly income (Peruvian sol)	0.99 (0.00)	0.99–1.00	1.00 (0.00)	0.99–1.01	0.99 (0.00)	0.99–1.00	1.00 (0.00)	0.99–1.00	0.99 (0.00)	0.99–1.00	0.99 (0.00)	0.99–1.00	0.99 (0.00)	0.99–1.00	0.99 (0.00)	0.99–1.00	0.99 (0.00)	0.99–1.00	**0.99 (0.01)**	**0.98-0.99**	1.00 (0.00)	0.99–1.01	0.99 (0.00)	0.99–1.00
Household infrastructure characteristics																								
Number of people sleeping in the household																								
≤ 5 (*n* = 44)	REF	REF	REF	REF	REF	REF	REF	REF	REF	REF	REF	REF	REF	REF	REF	REF	REF	REF	REF	REF	REF	REF	REF	REF
> 5 (*n* = 59)	1.60 (0.78)	0.63–4.04	**3.93 (2.55)**	**1.10-14.02**	1.20 (0.55)	0.49–2.94	1.54 (0.68)	0.65–3.66			0.91 (0.40)	0.39–2.16	0.85 (0.41)	0.33–2.16	0.72 (0.48)	0.19–2.67	0.94 (0.52)	0.32–2.76	0.44 (0.42)	0.07–2.89	0.77 (0.56)	0.18–3.24	0.31 (0.24)	0.07–1.39
Length of household tenancy (years)	–	–	–	–	–	–	–	–	–	–	–	–	–	–	–	–	–	–	–	–	–	–	–	–
Less than 1	REF	REF	REF	REF	REF	REF	REF	REF	REF	REF	REF	REF	REF	REF	REF	REF	REF	REF	REF	REF	REF	REF	REF	REF
Between 1 and 5	0.40 (0.33)	0.08–2.04	0.27 (0.26)	0.04–1.76	0.72 (0.54)	0.17–3.13	1.10 (0.85)	0.23–5.03	1.04 (0.74)	0.26–4.18	0.80 (0.64)	0.17–3.80	0.93 (0.77)	0.18–4.71	1.14 (1.49)	0.09–14.8	2.86 (2.73)	0.22–18.59	NA	NA	NA	NA	NA	NA
Between 5 and 10	1.38 (1.22)	0.24–7.82	0.16 (0.17)	0.02–1.20	1.00 (0.77)	0.22–4.48	0.89 (0.73)	0.18–4.42	2.54 (1.90)	0.59–10.97	0.50 (0.41)	0.10–2.51	1.02 (0.87)	0.19–5.47	0.30 (0.45)	0.02–5.71	0.57 (0.61)	0.06–4.69	NA	NA	NA	NA	NA	NA
Between 10 and 20	0.33 (0.29)	0.57–1.89	0.14 (0.16)	0.01–1.38	0.47 (0.39)	0.09–2.42	0.76 (0.66)	1.14–4.15	1.22 (0.96)	0.26–5.66	0.41 (0.36)	0.07–2.31	2.93 (2.58)	0.52–16.43	1.00 (1.45)	0.06–17.07	2.74 (2.81)	0.37–20.43	NA	NA	NA	NA	NA	NA
More than 20	**0.14 (0.12)**	**0.03-0.75**	0.12 (0.14)	0.01–1.14	0.52 (0.42)	0.11–2.51	0.78 (0.65)	0.56–3.14	0.98 (0.75)	0.22–4.41	0.47 (0.39)	0.09–2.44	2.11 (1.86)	0.37–11.88	3.14 (4.08)	0.25–40.00	1.14 (1.19)	0.15–8.88	NA	NA	NA	NA	NA	NA
Table material	–	–	–	–	–	–	–	–	–	–	–	–	–	–	–	–	–	–	–	–	–	–	–	–
Wood	–	–	REF	REF	–	–	REF	REF	–	–	REF	REF	–	–	REF	REF	–	–	REF	REF	–	–	REF	REF
Non-wood	–	–	**0.15 (0.13)**	**0.03-0.82**	–	–	**0.27 (0.14)**	**0.10-0.76**	–	–	0.39 (0.20)	0.14–1.07	–	–	0.24 (0.25)	0.03–1.83	–	–	0.16 (0.23)	0.01–2.74	–	–	0.62 (0.61)	0.09–4.28
Floor material	–	–	–	–	–	–	–	–	–	–	–	–	–	–	–	–	–	–	–	–	–	–	–	–
Unimproved	REF	REF	–	–	REF	REF	–	–	REF	REF	–	–	REF	REF	–	–	REF	REF	–	–	REF	REF	–	–
Improved	0.45 (0.23)	0.16–1.25	–	–	0.60 (0.27)	0.25–1.46	–	–	0.57 (0.25)	0.24–1.34	–	–	0.71 (0.33)	0.28–1.78	–	–	0.53 (0.30)	0.18–1.59	–	–	5.62 (4.21)	0.05–3.53	–	–
Wall material	–	–	–	–	–	–	–	–	–	–	–	–	–	–	–	–	–	–	–	–	–	–	–	–
Cement	REF	REF	REF	REF	REF	REF	REF	REF	REF	REF	REF	REF	REF	REF	REF	REF	REF	REF	REF	REF	REF	REF	REF	REF
Other	2.67 (1.84)	0.69–10.34	3.18 (2.56)	0.66–15.40	2.05 (1.27)	0.61–6.90	2.65 (1.61)	0.81–8.74	3.41 (2.12)	1.01–11.60	2.51 (1.51)	0.78–8.12	0.22 (0.16)	0.05–0.94	0.39 (0.41)	0.05–3.12	0.53 (0.30)	0.18–1.59	7.18 (9.44)	0.54–94.54	0.88 (0.87)	0.13–6.11	0.52 (0.56)	0.06–4.24
Hygiene characteristics																								
Household treating drinking water	–	–	–	–	–	–	–	–	–	–	–	–	–	–	–	–	–	–	–	–	–	–	–	–
No	REF	REF	REF	REF	REF	REF	REF	REF	REF	REF	REF	REF	REF	REF	REF	REF	REF	REF	REF	REF	REF	REF	REF	REF
Yes	1.39 (0.65)	0.55–3.50	2.39 (1.49)	0.71–8.09	**3.62 (1.71)**	**1.44-9.12**	**2.46 (1.08)**	**1.04-5.79**	1.56 (0.68)	0.66–3.68	**2.67 (1.19)**	**1.12-6.39**	0.65 (3.02)	1.82–4.72	2.21 (1.52)	0.57–8.55	1.95 (1.11)	0.64–5.95	0.31 (0.28)	0.05–1.78	1.34 (0.94)	0.34–5.29	0.34 (0.26)	0.08–1.51
Water source	–	–	–	–	–	–	–	–	–	–	–	–	–	–	–	–	–	–	–	–	–	–	–	–
Improved	REF	REF	REF	REF	REF	REF	REF	REF	REF	REF	REF	REF	REF	REF	REF	REF	REF	REF	REF	REF	REF	REF	REF	REF
Unimproved	5.90 (80.03)	0.41–84.88	5.73 (5.15)	0.98–33.41	3.30 (2.79)	0.63–17.28	1.36 (1.12)	0.27–6.84	1.29 (1.07)	0.25–6.61	1.48 (1.21)	0.30–7.31	0.86 (0.75)	0.16–4.75	3.57 (4.18)	0.36–35.54	**13.81 (14.68)**	**1.72-110.92**	0.33 (0.56)	0.01–8.94	6.44 (6.66)	0.85–48.95	NA	NA
Sanitation facility	–	–	–	–	–	–	–	–	–	–	–	–	–	–	–	–	–	–	–	–	–	–	–	–
Unimproved	REF	REF	REF	REF	REF	REF	REF	REF	REF	REF	REF	REF	REF	REF	REF	REF	REF	REF	REF	REF	REF	REF	REF	REF
Improved	0.43 (0.20)	0.17–1.08	1.31 (0.74)	0.44–3.94	1.98 (0.85)	0.85–4.60	0.81 (0.35)	0.35–1.87	1.20 (0.51)	0.52–2.74	0.64 (0.27)	0.28–1.46	1.11 (0.51)	0.45–2.73	0.84 (0.56)	0.22–3.13	0.49 (0.25)	0.17–1.35	1.31 (1.25)	0.20–8.47	0.31 (0.22)	0.07–1.25	2.53 (2.08)	0.50–12.68
Chickens in the household	–	–	–	–	–	–	–	–	–	–	–	–	–	–	–	–	–	–	–	–	–	–	–	–
No	REF	REF	REF	REF	REF	REF	REF	REF	REF	REF	REF	REF	REF	REF	REF	REF	REF	REF	REF	REF	REF	REF	REF	REF
Yes	**4.03 (2.02)**	**1.51-10.79**	**4.73 (2.93)**	**1.40-15.91**	**3.33 (1.55)**	**1.33-8.30**	1.32 (0.58)	0.56–3.13	1.53 (0.69)	0.64–3.68	0.89 (0.38)	0.38–2.07	2.42 (1.21)	0.91–6.45	2.35 (1.64)	0.59–9.26	1.63 (0.87)	0.58–4.63	1.31 (1.23)	0.21–8.29	2.68 (1.89)	0.67–10.71	3.09 (2.65)	0.57–16.60

OR = odds ratio.

### Microbial source tracking markers in surface samples.

#### Avian fecal marker Av4143.

Of the 104 floor samples, 49.0% (*n* = 51) of floors and 15.4% (*n* = 16) of tables (*P*-value < 0.001) were positive for the avian marker *Av4143*. The median log_10_ GCN/µL of Av4143 was 1.80 among floor samples and 1.33 among table samples (*P*-value < 0.001). The number of finished and unfinished floors, as well as wooden and non-wooden tables positive for *Av4143*, was not significantly different. Similarly, the median log_10_ GCN/µL of Av4143 among finished and unfinished floors, and wood and non-wood tables was not statistically different. Fifty percent of floor samples and 16.3% of table samples were classified as having a “high” quantity of Av4143 marker, whereas 17.3% of floors and 12.5% of table samples were “medium,” and 31.5% of floors and 71.2% of tables were “low” (*P*-value < 0.001). The OR of a floor sample being classified in the “high” tertile in comparison with the “middle” and low” tertiles was 3.70 (95% CI: 1.71–7.99; *P*-value = 0.001) among households who owned chickens in comparison to those who did not. This same statistically significant association was found among table samples scored for Av4143 (OR: 4.42; 95% CI: 1.70–11.49; *P*-value = 0.003). The OR of a table sample being classified in the “high” group in comparison to the “medium” and “low” groups was 3.32 (95% CI: 1.05–10.32; *P*-value = 0.041) among surfaces made of wood in comparison to material other than wood.

The multivariable model showed that the presence of chickens in the household (OR: 4.03; 95% CI: 1.51–10.79; *P*-value = 0.005), maternal age (OR: 0.93; 95% CI: 0.87–0.99; *P*-value = 0.044), and length of property tenancy (more than 20 years in comparison to less than 1 year, OR: 0.14; 95% CI: 0.03–0.75; *P*-value = 0.022) retained statistical significance among floor samples, whereas the presence of chickens (OR: 4.73; 95% CI: 1.40–15.91; *P*-value = 0.012) and table material (OR: 0.15; 95% CI: 0.03–0.82; *P*-value = 0.028) retained statistical significance among table samples, adjusted for all other covariates.

#### Avian mitochondrial markers CytB and ND5.

Fifty-nine percent (62/104) of floor samples and 67.3% (70/104) of table samples were positive for *CytB*, whereas 83.7% (87/104) floor samples and 77.9% (81/104) table samples were positive for *ND5*. A greater number of wood tables (84.0% [63/75]) were positive for the avian *ND5* marker, in comparison to non-wood tables (62.1% [18/29]) (*P*-value = 0.016). The median quantity of the avian markers *CytB* (wood: 4.31 log_10_ GCN/µL; non-wood 3.37 log_10_ GCN/µL; *P*-value = 0.038) and *ND5* (wood: 4.24 log_10_ GCN/µL; non-wood 3.43 log_10_ GCN/µL *P*-value = 0.010) was statistically different between wood and non-wood tables.

Of significance, wood tables in comparison to table surfaces made of material other than wood had higher odds of being classified as “high” tertile, for both the ND5 (OR: 3.11; 95% CI: 1.38–7.02; *P*-value = 0.006) and *CytB* markers (OR: 2.53; 95% CI: 1.12–5.68; *P*-value = 0.025). Within the final adjusted model for *ND5*, the presence of chickens in the household (OR: 3.33; 95% CI: 1.33–8.30; *P*-value = 0.10) and treatment of drinking water (OR: 3.62; 95% CI: 1.44–9.12; *P*-value = 0.006) retained statistical significance among floor samples, whereas table material (OR: 0.27; 95% CI: 0.10–0.76; *P*-value = 0.013) and treatment of drinking water (OR: 2.46; 95% CI: 1.04–5.79; *P*-value = 0.040) retained statistical significance among table samples. Within the final multivariable model for *CytB*, the OR of a floor sample being classified in the “high” tertile in comparison with the “middle” and “low” tertiles was 0.94 (95% CI: 0.89–0.99; *P*-value = 0.015), for every additional age of the primary caregiver. The effect of treatment of drinking water (OR: 2.67; 95% CI: 1.12–6.39; *P*-value = 0.027) also retained statistical significance among table samples.

#### Dog fecal marker *Bactcan*.

A total of 27.9% (29/104) of floors and 11.5% (12/104) of tables (*P*-value = 0.003) were positive for the dog fecal marker *Bactcan*. More unfinished floors (35.2% [19/54]) were positive than finished floors (20.0% [10/50]). Wood tables were 12.0% (9/75) positive and non-wood tables 10.3% (3/29). Once the quantities of *Bactcan* were categorized, 38.5% of floor samples and 11.5% of table were classified as having “high,” 8.7% of floors and 5.8% of tables were classified as “medium,” and 52.9% of floors and 82.7% of tables were classified as having “low” quantities of *Bactcan* marker (*P*-value < 0.001). From the univariate models, the odds of having a “high” *Bactcan* floor sample in comparison to a “medium” or “low” *Bactcan* floor sample were reduced by 70% (95% CI: 0.11–0.82; *P*-value = 0.020) among households with wall materials other than cement in comparison to those households with cement walls. No statistically significant associations were found between the categories of *Bactcan* and household infrastructure and socioeconomic characteristics.

#### Human fecal markers *Bachum* and *HF183*-Taqman

A total of 17.3% (18/104) of floors and 3.9% (4/104) of tables were positive for the human fecal marker *Bachum*. Eight of the floors were unfinished and 10 were finished. All four tables positive for the human fecal marker were made of wood. When categorized, 31.7% of floors and 8.7% of tables had a high *Bachum* burden, whereas 68.3% of floors and 91.3% of tables had a low *Bachum* burden (*P*-value < 0.001). Only one table and one floor were positive for the *HF183*-Taqman marker, which consisted of one finished floor and one wood table. In the final adjusted model, the odds of a table sample being classified in the “high” tertile in comparison with the “middle” and “low” tertiles were 0.81 (95% CI: 0.68–0.97; *P*-value = 0.020), for every additional age of the primary caregiver. Finally, in floor variables scored with the human *Bachum* fecal marker, the odds being classified in the “high” tertile in comparison with the “medium” and “low” tertiles were 13.81 (95% CI: 1.72–110.92; *P*-value = 0.014) among samples from households with unimproved water sources in comparison to households with improved water sources, holding all other variables constant. This point estimate should be interpreted with caution, given the small sample size associated with the model and associated high CIs.

### Microbial source tracking markers in toy samples.

A total of 55 toys were given to a child and collected within 24 hours. Avian fecal contamination exceeded microbial contamination from other sources: *ND5* (avian exposure) 41.8% (23/55), *CytB* (avian exposure) 20.0% (11/55), *Av4143* (avian feces) 7.3% (4/55), and *BacHum* (human feces) 3.6% (2/55). All toy samples were negative for *HF183*-Taqman (human feces), *Bactcan* (canine feces), *LA35* (avian feces), and *Pig2Bac* (swine feces). Of the 40 households with positive toys, 15.0% (6/40) had negative corresponding table or floor samples.

### Association between *Campylobacter* spp. detection and MST markers.

A total of 60.6% (63/104) floor surface samples (43 unfinished and 20 finished; *P*-value < 0.001) and 18.3% (19/104) table surface samples (16 wood and 3 non-wood; *P*-value = 0.193) were positive for *Campylobacter* spp. (*16S* gene; *P*-value < 0.001). Among floor samples, the OR of detecting *Campylobacter* spp. was 29.34 (95% CI: 7.61–113.17; *P*-value < 0.001) among samples positive for Av4143, adjusting for the presence of chickens and floor material. Among table samples, the OR of detecting *Campylobacter* spp. was 29.49 (95% CI: 6.71–129.55; *P*-value < 0.001) among samples positive for Av4143, adjusting for the presence of chickens and table material. Similarly, there was a higher odds of detecting *Campylobacter* spp. in floor and table samples if samples were positive for *CytB* (floors: OR: 12.61; 95% CI: 4.11–38.68; *P*-value < 0.001; tables: OR: 10.72; 95% CI: 1.36–84.75; *P*-value = 0.025) and among floor samples if they were positive for *ND5* (OR: 10.25; 95% CI: 2.43–43.32; *P*-value = 0.002). The OR of detecting *Campylobacter* spp. in floor samples was 25.25 among samples positive for the human *Bachum* (95% CI: 2.94–216.81; *P*-value = 0.003). A multivariable model adjusting for the presence of all markers, as well as the presence of chickens in the household and the floor material, showed that the odds of having a *Campylobacter* spp.–positive sample increased by 11.71 (95% CI: 2.59–53.09; *P*-value = 0.001) among floor samples positive for the *Av4143* marker, and decreased by 0.11 (95% CI: 0.03–0.39; *P*-value = 0.001) if floors were finished in comparison to unfinished floors. All other markers showed no association with the odds of detecting *Campylobacter* spp. in a floor sample in models adjusted for all markers, suggesting that identifiable exposures rather than universal community-wide contamination drove household levels of risk for campylobacteriosis. Similarly, among table samples, the OR of having a *Campylobacter* spp.–positive sample was 21.74 (95% CI: 4.62–101.38; *P*-value < 0.001) among samples positive for *Av4143* in comparison to negative samples, adjusting for all other markers, presence of chickens, and table material. No other covariate was associated with the presence of *Campylobacter* spp. from a table surface sample.

## DISCUSSION

In three peri-urban communities of Iquitos, Peru, the prevalence of animal fecal material, most notably avian-derived contamination, exceeded that of human fecal material in structured sampling of household surfaces. Specifically, 76.9% household surfaces were positive for any fecal MST marker, 75.0% of households were positive for animal feces, and 20.2% were positive for human feces. Detection of avian markers was associated with the material of the surface of the table sampled, presence of chickens in the household, maternal age, and length of property tenance, whereas detection of human markers was associated with unimproved water source. This study further demonstrated that detection of viable *Campylobacter* spp. was associated with detection of avian marker *Av4143*, even after adjustment for the presence of chickens and the presence of other markers. Overall, this study strongly suggests that animal sources are important to fecal contamination in households in a tropical low-resource community.

Chickens are highly ubiquitous animals in communities in the Peruvian Amazon, given that poultry is the main source of animal protein besides fish, and are commonly raised as an alternative source of income or as pets. Chickens are seldom corralled, and there is no physical barrier that prohibits their entrance to the living or cooking spaces.

The odds of detecting high quantities of *Av4143*, *CytB*, and *ND5* were strongly associated with the presence of chickens in the household, corroborating the utility of these MST markers in this particular setting. In addition, having a positive *Av4143* floor or table sample had a strong association with *Campylobacter* spp.–positive surface samples. Although chickens are considered a risk factor for *Campylobacter* spp. infections, we do not advocate discouraging the practice of chicken rearing. On the contrary, chickens and eggs are an important nutritional source of protein and iron. However, these data suggest the potential importance of mitigation strategies to reduce exposure to avian fecal material, and future studies will be needed to identify strategies that are effective but that do not have unintended consequence for economic stability of households, animal health, or human health. In fact, studies in Peru and Ethiopia present evidence that suggests corralling chickens might increase the risk of *Campylobacter* spp. transmission and infection, potentially by affecting the ecology of *Campylobacter* spp. in the host or by increasing the degree of animal crowding and, therefore, overall concentration of fecal burden in a single location within the household.^42,43^ Results from this study indicate that a higher burden of avian exposure, as measured by *CytB* and *ND5*, is associated with wood tables than non-wood tables. This is one potential risk factor amenable to intervention that could be tested directly in future trials, given cooking tables made or covered of materials such as plastic or tiles are more frequently cleaned than non-wood tables (personal observation). Recent randomized clinical trials that aim to reduce the burden of enteric disease using water, hygiene, and sanitation practices have not been unsuccessful.^44–46^ Although reasons for these are multifaceted, some studies have already suggested the need to incorporate household infrastructure improvements to reduce the burden of fecal contamination. Among many, these include building cement floors, improved water distribution systems, and improved waste management systems. A recent call to for an “integrative management of animals, wash, sanitation, and hygiene” highlights the need for transformative Water, Sanitation, and Hygiene interventions.^47^ This study provides data to support the importance of addressing animal fecal waste within household environments and to introduce a One Health approach to water sanitation and hygiene research.

We found few samples positive for the human fecal markers *Bachum* and *HF183*-Taqman. However, among samples that were positive for *Bachum*, the odds of having high human fecal contamination were highly increased if the household had unimproved water. Although human MST markers have been most frequently used in resource-poor environments, to our knowledge, they have not been applied to household surface samples.^20,21,48,49^ *Pig2Bac* and *LA35* markers were not detected in any surface sample. The lack of positive samples to *Pig2Bac* is not surprising, given that few households own and raise pigs. The absence of positive LA35 markers is attributed to the particularly low sensitivity of this marker.^29^

Limitations of this study include the fact that the performance of MST markers is setting specific and requires a previous validation step. In addition, new human MST markers that have higher degrees of sensitivity and specificities are required. Given the emerging microbiome research, the development of markers taking into consideration age-specific features of fecal microbiota, age-specific *Bifidobacterium*, and other fastidious members of the flora could significantly enhance our understanding of the source of human fecal contamination. It is important to note that the lack of positive surface samples to certain MST outcomes may have prevented the precise estimation of associations. However, results show a consistent positive association between the presence of *Campylobacter* and avian MST markers, despite the stated sample size limitation.

Future studies should include water samples along with surface samples as a further method of comparison, as well as samples from toilet areas and human hands, as hand-to-mouth ingestion of avian fecal material has been shown previously.^50^ Comparing the burden of MST markers in these additional sampling environments will help elucidate the degree of animal and human fecal waste among potential transmission pathways that exist in these environments. Finally, the isolation of viable *Campylobacter* from surface samples would have strengthened our findings.

Human and avian fecal markers as well as avian exposure markers were detected among the 55 toys sampled. The high degree of positivity to avian and human markers suggests that toys serve as a relevant sentinel of animal fecal exposure and that both avian and human feces are frequently in contact with household members.

## CONCLUSION

Avian fecal material is highly prevalent in floor and cooking spaces of households located in peri-urban, low-resource tropical communities. There is a need to include animal fecal waste in interventions that target water, hygiene, and sanitation aiming to reduce the burden of enteric disease and environmental enteropathy. However, care should be taken to do this in a way that recognizes the key food security role chickens and eggs play in pediatric and adult populations living in resource-poor settings and should be used to shape, rather than discourage, domestic animal husbandry practices. This study adds to mounting evidence for the need to treat the domestic environment as a single entity using a One Health strategy, and to support simple interventions to decrease exposure to avian fecal material and potentially pathogenic bacteria such as the use of easy-to-clean plastic surfaces on cooking tables.

## Supplemental tables

Supplemental materials
