# Supplementary material for: Associations among Household Animal Ownership, Infrastructure, and Hygiene Characteristics with Source Attribution of Household Fecal Contamination in Peri-Urban Communities of Iquitos, Peru
Source: Am J Trop Med Hyg. 2020 Nov 2;104(1):372–81. doi: 10.4269/ajtmh.20-0810 (PMC7790101; doi:10.4269/ajtmh.20-0810)
Supplement: Supplementary file 1 [file tpmd200810.SD1.pdf]

Supplemental Table 1. Target Gene, Host, Primers and Probes, Sensitivity, Specificity, Positive Predictive Value, Negative Predictive Value and Accuracy of Eight Microbial Source Tracking (MST) Markers used for Source Attribution

Marker	Target	Host	Primers and Probes		Sensitivity	Specificity	Positive Predictive Value	Negative Predictive Value	Accuracy
LA35	*Brevibacterium avium*	Chickens	LA35F	5′-ACC GGA TAC GAC CAT CTG C-3′	23.10%	100.00%	100.00%	89.40%	89.70%
			LA35R	5′-TCC CCA GTG TCA GTC ACA GC-3′					
			Probe	5′-FAM-CAG CAG GGA AGA AGC CTT CGG GTG ACG GTA-BHQ1-3′					
ND5	Mitochondrial DNA (NADH dehydrogenase subunit 5)	Chickens and Ducks	ND5-F	5'-ACCTCCCCCAACTAGC-3'	69.60%	75.70%	47.10%	78.10%	66.00%
			ND5-R	5'-TTGCCAATGGTTAGGCAGGAG-3'					
			ND5-P	5'-FAM-TCAACCCATGCCTTCTT-NFQ-MGB-3'					
CytB	Mitochondrial DNA (cytochrome b)	Chickens and Ducks	cytb-F	5'-AAATCCCACCCCCTACTAAAAATAAT-3'	87.00%	82.40%	60.60%	95.30%	83.50%
			cytb-R	5'-CAGATGAAGAAGAATGAGGCG-3'					
			cytb-P	5'-FAM-ACAACTCCCTAATCGACCT-NFQ-MGB-3′					
Av4143	*Lactobacillus* spp.	Domestic Birds and Waterfoul	Av4143F	5'-TGCAAGTCGAACGAGGATTTCT-3'	72.70%	87.50%	75.00%	86.20%	82.50%
			Av4143R	5'-TCACCTTGGTAGGCCGTTACC-3'					
			Av4143P	5'-FAM-AGGTGGTTTTGCTATCGCTTT-BHQplus-3′					
BacCan	*Bacteroidales*	Dogs	BactCan545f1	5'-GGAGCGCAGACGGGTTTT-3'	100.00%	47.40%	19.60%	100.00%	53.40%
			Uni/Cow690r1	5'-CAATCGGAGTTCTTCGTGATATCTA-3'					
			Uni/Cow 690r2	5'-AATCGGAGTTCCTCGTGATATCTA-3'					
			Uni/Cow 656p	5'-FAM-TGGTGTAGCGGTGAAA-MGB-3'					
Pig2Bac	*Bacteroidales*	Pigs	Pig-2-Bac41F	5'-GCATGAATTTAGCTTGCTAAATTTGAT-3'	100.00%	88.50%	50.00%	100.00%	89.70%
			Pig-2-Bac163Rv	5'-ACCTCATACGGTATTAATCCGC-3'					
			Pig-2Bac113	5'-VIC-TCCACGGGATAGCC-NFQ-MGB-3'					
HF183-Taq	*Bacteroidales*	Humans	HF183f	5'-ATCATGAGTTCACATGTCCG-3'	76.70%	67.60%	50.00%	87.30%	70.30%
			BthetR1	5'-CGTAGGAGTTTGGACCGTGT-3'					
			BthetP1	5'-FAM-CTGAGAGGAAGGTCCCCCACATTGGA-TAMRA-3'					
BacHum	*Bacteroidales*	Humans	BacHum160Fw	5'-TGAGTTCACATGTCCGCATGA-3'	80.00%	66.20%	50.00%	88.70%	70.30%
			BacHum241Rv	5'-CGTTACCCCGCCTACTATCTAATG-3'					
			BacHum193Probe	5'-FAM-TCCGGTAGACGATGGGGATGCGTT-TAMRA-3'					

Supplemental Table 2: *Campylobacter* spp. primers and probe sequences.

Target	Primers	Probe
16S	16S_Fw 5’- CAC GTG CTA CAA TGG CAT AT -3’<br>16S_Rv 5’- GGC TTC ATG CTC TCG AGT T -3’	5’- /56-FAM/CAG AGA ACA /ZEN/ ATC CGA ACT GGG ACA /3IABkFQ/ -3’
cadF	cadF_Fw: 5’- CTG CTA AAC CAT AGA AAT AAA ATT TCT CAC -3’;<br>cadF_Rv: 5’- CTT TGA AGG TAA TTT AGA TAT GGA TAA TCG -3’	5’ -/56-JOEN/CAT TTT GAC /ZEN/ GAT TTT TGG CTT GA/3IABkFQ/ -3’

Supplemental Table 3. Tertiles of contamination of (A) floor and table samples, (B) unfinished and finished floors, and (C) wood and non-wood samples obtained from households in three communities of Iquitos, Peru, as measured by microbial source tracking markers.

MST Marker	Floors (n=104)			Tables (n=104)			p-value
	Low	Medium	High	Low	Medium	High	
Av4143	32.7% (34/104)	17.3% (18/104)	50% (52/104)	71.2% (74/104)	12.5% (13/104)	16.3% (17/104)	**<0.001**
CytB	34.6% (36/104)	37.5% (39/104)	27.9% (29/104)	32.7% (34/104)	28.8% (30/104)	38.5% (40/104)	0.273
ND5	36.5% (38/104)	36.5% (38/104)	26.95% (28/104)	30.8% (32/104)	29.8% (31/104)	39.4% (41/104)	0.108
Bactcan	52.9% (55/104)	8.7% (9/104)	38.5% (40/104)	82.7% (86/104)	5.8% (6/104)	11.5% (12/104)	**<0.001**
Bachum	68.3% (71/104)	NA	31.7% (33/104)	91.3% (95/104)	NA	8.7% (9/104)	**<0.001**
HF-183 Taqman	80.8% (84/104)	NA	19.2% (20/104)	90.4% (94/104)	NA	9.6% (10/104)	0.074

MST Marker	Unfinished(n=54)			Finished (n=50)			p-value
	Low	Medium	High	Low	Medium	High	
Av4143	27.8% (15/54)	16.7% (9/54)	55.6% (30/54)	38.0% (19/50)	18.0% (9/50)	44.0% (22/50)	0.461
CytB	27.8% (15/54)	46.3% (25/54)	25.9% (14/54)	42.0% (21/50)	28.0% (14/50)	30.0% (15/50)	0.136
ND5	29.6% (16/54)	44.4% (24/54)	25.9% (14/54)	44.0% (22/50)	28.0% (14/50)	28.0% (14/50)	0.180
Bactcan	46.3% (25/54)	7.4% (4/54)	46.3% (25/54)	60.0% (30/50)	10.0% (5/50)	30.0% (15/50)	0.245
Bachum	64.8% (35/54)	NA	35.2% (19/54)	72.0% (36/50)	NA	28.0% (14/50)	0.432
HF-183 Taqman	89.9 (48/54)	NA	11.1% (6/54)	72.0% (36/50)	NA	28.0% (14/50)	**0.026**

MST Marker	Wood (n=75)			Non-Wood (n=29)			p-value
	Low	Medium	High	Low	Medium	High	
Av4143	65.3% (49/75)	14.7% (11/75)	20.0% (15/75)	86.2% (25/29)	6.9% (2/29)	6.9% (2/29)	0.106
CytB	26.7% (20/75)	29.3% (22/75)	44.0% (33/75)	48.3% (14/29)	27.6% (8/29)	24.1% (7/29)	0.076
ND5	24.0% (18/75)	29.3% (22/75)	46.7% (35/75)	48.3% (14/29)	20.7% (6/29)	31.0% (9/29)	**0.022**
Bactcan	80.0% (60/75)	6.7% (5/75)	13.3% (10/75)	89.7% (26/29)	3.5% (1/29)	6.9% (2/29)	0.608
Bachum	89.3% (67/75)	NA	10.7% (8/75)	96.6% (28/29)	NA	3.5% (1/29)	0.240
HF-183 Taqman	89.3% (67/75)	NA	10.7% (8/75)	93.1% (27/29)	NA	6.9% (2/29)	0.434

All samples were negative for *LA35* and *Pig2Bac* microbial source tracking markers.

Supplemental Table 4. Unadjusted associations between female head of household, infrastructure and hygiene characteristics and the change in log(10) gene copy number (GCP) of each microbial source tracking marker among floor samples

	**AV4143**			**ND5**			**CYTB**			**BactCan**			**BacHum**			**HF183-Taqman**		
	Odds Ratio (SD)	95% CI	p-value	Odds Ratio (SD)	95% CI	p-value	Odds Ratio (SD)	95% CI	p-value	Odds Ratio (SD)	95% CI	p-value	Odds Ratio (SD)	95% CI	Odds Ratio (SD)	β (SD)	95% CI	p-value
**Female Head of Household Characteristics**																		
Age (years)	**0.95 (0.02)**	**[0.91-0.99]**	**0.039**	0.97 (0.02)	[0.93-1.01]	0.094	0.96 (0.02)	[0.93-1.00]	0.08	1.04 (0.02)	[0.99-1.08]	0.101	1.00 (0.02)	[0.96-1.05]	0.914	0.98 (0.03)	[0.92-1.04]	0.42
Maternal Education (years)	1.02 (0.07)	[0.90-1.18]	0.681	0.98 (0.07)	[0.86-1.13]	0.802	0.95 (0.06)	[0.82-1.09]	0.427	0.99 (0.07)	[0.89-1.14]	0.848	0.99 (0.08)	[0.84-1.16]	0.866	0.96 (0.09)	[0.80-1.15]	0.656
Age of First Pregnancy (years)	1.02 (0.05)	[0.93-1.12]	0.691	1.10 (0.05)	[0.93-1.11]	0.785	0.98 (0.04)	[0.89-1.07]	0.662	1.01 (0.05)	[0.92-1.10]	0.904	0.99 (0.05)	[0.90-1.10]	0.922	1.03 (0.06)	[0.91-1.16]	0.648
Average Monthly Income (US Dollars)	0.99 (0.00)	[0.99-1.00]	0.893	0.99 (0.00)	[0.99-1.00]	0.413	0.99 (0.00)	[0.99-1.00]	0.789	0.99 (0.00)	[0.99-1.00]	0.068	0.99 (0.00)	[0.99-1.00]	0.718	0.99 (0.00)	[0.99-1.00]	0.444
**Household Infrastructure Characteristics**																		
Number of people sleeping in household																		
≤ 5 (n=44)	[REF]	[REF]	[REF]	[REF]	[REF]	[REF]	[REF]	[REF]	[REF]	[REF]	[REF]	[REF]	[REF]	[REF]	[REF]	[REF]	[REF]	[REF]
> 5 (n=59)	1.68 (0.64)	[0.80-3.53]	0.172	1.60 (0.58)	[0.77-3.25]	0.214	1.24 (0.45)	[0.61-2.54]	0.556	0.90 (0.34)	[0.42-1.90]	0.774	0.78 (0.34)	[0.34-1.81]	0.567	1.03 (0.53)	[0.38-2.83]	0.952
Length of Household Tenancy																		
Less than 1 year	[REF]	[REF]	[REF]	[REF]	[REF]	[REF]	[REF]	[REF]	[REF]	[REF]	[REF]	[REF]	[REF]	[REF]	[REF]	[REF]	[REF]	[REF]
Between 1 and 5 years	0.43 (0.30)	[0.11-1.66]	0.222	1.02 (0.64)	[0.30-3.51]	0.975	1.28 (0.79)	[0.39-4.28]	0.684	1.10 (0.80)	[0.27-4.55]	0.886	3.75 (3.24)	[0.69-20.38]	0.126	1.67 (1.48)	[0.29-9.52]	0.566
Between 5 and 10 years	1.62 (0.30)	[0.35-7.53]	0.538	1.62 (1.05)	[0.46-5.75]	0.456	2.82 (1.81)	[0.81-9.90]	0.104	1.60 (1.19)	[0.37-6.91]	0.527	1.88 (1.71)	[0.31-11.17]	0.490	1.88 (1.71)	[0.31-11.17]	0.490
Between 10 and 20 years	0.38 (0.29)	[0.08-1.73]	0.210	0.73 (0.53)	[0.18-3.00]	0.661	1.21 (0.84)	[0.31-4.73]	0.787	3.74 (0.3.02)	[0.77-18.14]	0.101	3.75 (3.54)	[0.59-23.87]	0.162	1.36 (1.38)	[0.19-9.91]	0.759

More than 20 years	**0.18 (0.13)**	**[0.05-0.74]**	**0.017**	0.64 (0.41)	[0.18-2.23]	0.481	1.16 (0.72)	[0.35-3.90]	0.806	1.73 (1.23)	[0.42-6.99]	0.444	1.67 (1.48)	[0.29-9.52]	0.566	0.38 (0.41)	[0.05-3.11]	0.371
Floor Material																		
Unfinished	[REF]	[REF]	[REF]	[REF]	[REF]	[REF]	[REF]	[REF]	[REF]	[REF]	[REF]	[REF]	[REF]	[REF]	[REF]	[REF]	[REF]	[REF]
Finished	0.63 (0.24)	[0.30-1.31]	0.214	0.73 (0.27)	[0.36-1.49]	0.387	0.77 (0.28)	[0.37-1.57]	0.474	0.54 (0.21)	[0.25-1.15]	0.112	0.72 (0.30)	[0.31-1.65]	0.432	3.11 (1.67)	[1.09-8.89]	0.034
Wall Type																		
Cement	[REF]	[REF]	[REF]	[REF]	[REF]	[REF]	[REF]	[REF]	[REF]	[REF]	[REF]	[REF]	[REF]	[REF]	[REF]	[REF]	[REF]	[REF]
Other	0.89 (0.40)	[0.37-2.13]	0.796	0.99 (0.45)	[0.41-2.43]	0.993	1.60 (0.71)	[0.67-3.84]	0.29	**0.30**	**[0.11-0.82]**	**0.020**	0.65 (0.35)	[0.23-1.84]	0.421	0.53 (0.36)	[0.14-1.99]	0.346
**Hygiene Characteristics**																		
Household treating drinking water																		
No	[REF]	[REF]	[REF]	[REF]	[REF]	[REF]	[REF]	[REF]	[REF]	[REF]	[REF]	[REF]	[REF]	[REF]	[REF]	[REF]	[REF]	[REF]
Yes	1.27 (0.48)	[0.60-2.69]	0.531	2.07 (0.79)	[0.98-4.39]	0.058	1.36 (0.51)	[0.66-2.82]	0.408	0.67 (0.26)	[0.31-1.46]	0.316	1.68 (0.75)	[0.70-4.05]	0.246	1.20 (0.62)	[0.43-3.33]	0.724
Water Source																		
Improved	[REF]	[REF]	[REF]	[REF]	[REF]	[REF]	[REF]	[REF]	[REF]	[REF]	[REF]	[REF]	[REF]	[REF]	[REF]	[REF]	[REF]	[REF]
Unimproved	4.59 (3.72)	[0.94-22.44]	0.06	**3.56 (2.23)**	**[1.04-12.13]**	**0.042**	1.98 (1.21)	[0.60-6.59]	0.263	1.11 (0.71)	[0.32-3.89]	0.872	**7.25 (5.20)**	**[1.78-29.53]**	**0.006**	**6.77 (4.55)**	**[1.82-25.24]**	**0.004**
Sanitation Facility																		
Unimproved	[REF]	[REF]	[REF]	[REF]	[REF]	[REF]	[REF]	[REF]	[REF]	[REF]	[REF]	[REF]	[REF]	[REF]	[REF]	[REF]	[REF]	[REF]
Improved	0.56 (0.21)	[0.27-1.16]	0.120	1.40 (0.51)	[0.68-2.84]	0.358	1.02 (0.37)	[0.50-2.07]	0.964	0.87 (0.33)	[0.41-1.84]	0.725	0.68 (0.29)	[0.29-1.56]	0.359	0.64 (0.32)	[0.24-1.71]	0.37
Chickens in Household																		
No	[REF]	[REF]	[REF]	[REF]	[REF]	[REF]	[REF]	[REF]	[REF]	[REF]	[REF]	[REF]	[REF]	[REF]	[REF]	[REF]	[REF]	[REF]
Yes	**3.70 (1.45)**	**[1.71-7.99]**	**0.001**	**3.29 (1.25)**	**[1.56-6.92]**	**0.002**	1.94 (0.71)	[0.94-3.98]	0.072	1.62 (0.62)	[0.76-3.43]	0.211	2.10 (0.90)	[0.91-4.88]	0.084	2.36 (1.22)	[0.85-6.51]	0.097

Supplemental Table 5. Unadjusted associations between female head of household, infrastructure and hygiene characteristics and the change in log(10) gene copy number (GCP) of each microbial source tracking marker among table samples

	**AV4143**			**ND5**			**CYTB**			**BactCan**			**BacHum**			**HF183-Taqman**		
	Odds Ratio (SD)	95% CI	p-value	Odds Ratio (SD)	95% CI	p-value	Odds Ratio (SD)	95% CI	p-value	Odds Ratio (SD)	95% CI	p-value	Odds Ratio (SD)	95% CI	p-value	Odds Ratio (SD)	95% CI	p-value
**Female Head of Household Characteristics**																		
Age (years)	0.98 (0.02)	[0.93-1.03]	0.334	0.98 (0.02)	[0.94-1.01]	0.196	0.97 (0.02)	[0.93-1.00]	0.075	1.03 (0.03)	[0.98-1.08]	0.287	0.92 (0.05)	[0.83-1.02]	0.105	1.03 (0.03)	[0.97-1.10]	0.319
Maternal Education (years)	1.00 (0.08)	[0.85-1.18]	0.967	0.99 (0.07)	[0.86-1.13]	0.832	0.99 (0.07)	[0.86-1.13]	0.830	1.06 (0.11)	[0.86-1.30]	0.607	1.04 (0.14)	[0.79-1.36]	0.788	0.87 (0.10)	[0.69-1.10]	0.248
Age of First Pregnancy (years)	0.97 (0.05)	[0.88-1.08]	0.572	0.97 (0.04)	[0.89-1.05]	0.414	0.94 (0.04)	[0.87-1.03]	0.178	0.99 (0.06)	[0.88-1.13]	0.910	1.00 (0.09)	[0.95-1.19]	0.974	0.95 (0.08)	[0.80-1.12]	0.527
Average Monthly Income (US Dollars)	1.00 (0.00)	[0.99-1.00]	0.202	0.99 (0.00)	[0.99-1.00]	0.878	0.99 (0.00)	[0.99-1.00]	0.771	0.99 (0.00)	[0.99-1.00]	0.127	0.99 (0.00)	[0.99-1.00]	0.142	0.99 (0.00)	[0.99-1.00]	0.571
**Household Infrastructure Characteristics**																		
Number of people sleeping in household																		
<=5 (n=44)	[REF]	[REF]	[REF]	[REF]	[REF]	[REF]	[REF]	[REF]	[REF]	[REF]	[REF]	[REF]	[REF]	[REF]	[REF]	[REF]	[REF]	[REF]
>5 (n=59)	**3.16 (1.49)**	**[1.26-7.96]**	**0.014**	1.45 (0.54)	[0.70-2.99]	0.317	0.91 (0.34)	[0.44-1.88]	0.803	0.55 (0.29)	[0.20-1.54]	0.774	0.93 (0.65)	[0.23-3.67]	0.913	0.46 (0.31)	[0.121-1.74]	0.254
Length of Household Tenancy																		
Less than 1 year	[REF]	[REF]	[REF]	[REF]	[REF]	[REF]	[REF]	[REF]	[REF]	[REF]	[REF]	[REF]	[REF]	[REF]	[REF]	[REF]	[REF]	[REF]
Between 1 and 5 years	0.64 (0.48)	[0.14-2.78]	0.551	1.33 (0.68)	[0.35-5.13]	0.676	1.00 (0.07)	[0.25-3.98]	1.000	1.53 (1.81)	[0.15-15.61]	0.720	0.87 (0.81)	[0.14-5.40]	0.881	1.47E+07 (4.38E+10)	NA	0.996
Between 5 and 10 years	0.74 (0.57)	[0.16-3.34]	0.698	0.96 (0.66)	[0.25-3.72]	0.952	0.62 (0.44)	[0.15-2.52]	0.507	0.81 (2.05)	[0.07-10.14]	0.872	4.14E-08	NA	0.993	1.00 (3561.53)	NA	1.000
Between 10 and 20 years	0.25 (0.25)	[0.03-1.73]	0.16	0.68 (0.52)	[0.15-3.09]	0.615	0.44 (0.35)	[0.90-2.12]	0.305	1.53 (1.99)	[0.12-19.65]	0.745	4.14E-08	NA	0.995	6.80E+06 (2.02E+10)	NA	0.996

More than 20 years	0.40 (0.31)	[0.09-1.80)	0.232	0.86 (0.58)	[0.23-3.23]	0.827	0.62 (0.44)	[0.16-2.44]	0.498	3.5 (3.94)	[0.39-31.77]	0.265	0.29 (0.32)	[0.04-2.45]	0.259	1.84E+07 (5.48E+10)	NA	0.996
Table Material																		
Wood	[REF]	[REF]	[REF]	[REF]	[REF]	[REF]	[REF]	[REF]	[REF]	[REF]	[REF]	[REF]	[REF]	[REF]	[REF]	[REF]	[REF]	[REF]
Non-Wood	**3.32 (1.95)**	**[1.05-10.52]**	**0.041**	**3.11 (1.29)**	**[1.38-7.02]**	**0.006**	**2.53 (1.05)**	**[1.12-5.68]**	**0.025**	2.16 (1.45)	[0.58-8.08]	0.253	3.34 (3.63)	[0.40-27.99]	0.266	1.61 (1.33)	[0.32-8.08]	0.562
Wall Material																		
Cement	[REF]	[REF]	[REF]	[REF]	[REF]	[REF]	[REF]	[REF]	[REF]	[REF]	[REF]	[REF]	[REF]	[REF]	[REF]	[REF]	[REF]	[REF]
Other	1.41 (0.67)	[0.56-3.58]	0.471	1.38 (0.58)	[0.60-3.16]	0.446	1.34 (0.57)	[0.58-3.10]	0.484	0.55 (0.37)	[0.15-2.07]	0.376	1.55 (1.16)	[0.36-6.76]	0.548	0.73 (0.60)	[0.14-3.67]	0.702
**Hygiene Characteristics**																		
Household treating drinking water																		
No	[REF]	[REF]	[REF]	[REF]	[REF]	[REF]	[REF]	[REF]	[REF]	[REF]	[REF]	[REF]	[REF]	[REF]	[REF]	[REF]	[REF]	[REF]
Yes	1.50 (0.69)	[0.61-3.68]	0.379	1.97 (0.74)	[0.95-4.10]	0.069	**2.32 (0.88)**	**[1.10-4.88]**	**0.027**	1.31 (0.71)	[0.45-3.79]	0.625	0.47 (0.33)	[0.12-1.85]	0.279	0.59 (0.40)	[0.16-2.19]	0.434
Water Source																		
Improved	[REF]	[REF]	[REF]	[REF]	[REF]	[REF]	[REF]	[REF]	[REF]	[REF]	[REF]	[REF]	[REF]	[REF]	[REF]	[REF]	[REF]	[REF]
Unimproved	**3.48 (2.14)**	**[1.04-11.61]**	**0.043**	1.00 (0.58)	[0.33-3.09]	1.000	0.70 (0.40)	[0.23-2.16]	0.538	1.16 (0.97)	[0.23-5.91]	0.855	1.06 (1.18)	[0.12-9.39]	0.957	1.68E-07 (0.00)	NA	0.994
Sanitation Facility																		
Unimproved	[REF]	[REF]	[REF]	[REF]	[REF]	[REF]	[REF]	[REF]	[REF]	[REF]	[REF]	[REF]	[REF]	[REF]	[REF]	[REF]	[REF]	[REF]
Improved	1.55 (0.67)	[0.67-3.60]	0.308	0.75 (0.27)	[0.37-1.53]	0.434	0.61 (0.22)	[0.30-1.23]	0.169	0.83 (0.43)	[0.30-2.29]	0.713	1.33 (0.93)	[0.34-5.27]	0.683	1.04 (0.69)	[0.28-3.84]	0.949
Chickens in Household																		
No	[REF]	[REF]	[REF]	[REF]	[REF]	[REF]	[REF]	[REF]	[REF]	[REF]	[REF]	[REF]	[REF]	[REF]	[REF]	[REF]	[REF]	[REF]
Yes	**4.42 (2.15)**	**[1.70-11.49]**	**0.002**	1.49 (0.54)	[0.73-3.04]	0.274	1.00 (0.36)	[0.50-2.05]	0.983	1.46 (0.76)	[0.53-4.03]	0.470	1.28 (0.90)	[0.32-5.05]	0.728	1.00 (0.67)	[0.27-3.68]	1.000
